# Supplementary material for: From sequence to enzyme mechanism using multi-label machine learning
Source: BMC Bioinformatics. 2014 May 19;15:150. doi: 10.1186/1471-2105-15-150 (PMC4229970; doi:10.1186/1471-2105-15-150)
Supplement: Additional file 2 — Java code of ml2db. Additional file ml2db_code.tar.gz contains the Java source code to run the multi-label machine learning experiments and save the results to database. The code’s Javadoc is included. [file 1471-2105-15-150-S2.zip › additional file 2/ml2db/utils/doc/uk/ac/standrews/utils/main/Initialised.html]

Initialised


JavaScript is disabled on your browser.


- Overview
- Package
- Class
- Use
- Tree
- Deprecated
- Index
- Help

- Prev Class
- Next Class

- Frames
- No Frames

- All Classes

- Summary:
- Nested |
- Field |
- Constr |
- Method

- Detail:
- Field |
- Constr |
- Method


uk.ac.standrews.utils.main

## Class Initialised

- java.lang.Object
- - uk.ac.standrews.utils.main.Initialised

- All Implemented Interfaces:
  :   Initialisable

  ---

    

  ```
  public class Initialised
  extends java.lang.Object
  implements Initialisable
  ```

- - ### Field Summary

    Fields

    | Modifier and Type | Field and Description |
    | --- | --- |
    | `boolean` | `m_initialisationIsCorrect` |
  - ### Constructor Summary

    Constructors

    Constructor and Description

    `Initialised()`
  - ### Method Summary

    Methods

    | Modifier and Type | Method and Description |
    | --- | --- |
    | `boolean` | `initialisationIsCorrect()` |
    | `void` | `setInitialisation(boolean isCorrect)` |
    | `void` | `setInitialisationIsCorrect()` |
    | `void` | `setInitialisationNotCorrect()` |

    - ### Methods inherited from class java.lang.Object

      `equals, getClass, hashCode, notify, notifyAll, toString, wait, wait, wait`

- - ### Field Detail


    - #### m\_initialisationIsCorrect

      ```
      public boolean m_initialisationIsCorrect
      ```
  - ### Constructor Detail


    - #### Initialised

      ```
      public Initialised()
      ```
  - ### Method Detail


    - #### initialisationIsCorrect

      ```
      public boolean initialisationIsCorrect()
      ```

      **Specified by:**
      :   `initialisationIsCorrect` in interface `Initialisable`


    - #### setInitialisation

      ```
      public void setInitialisation(boolean isCorrect)
      ```

      **Specified by:**
      :   `setInitialisation` in interface `Initialisable`


    - #### setInitialisationIsCorrect

      ```
      public void setInitialisationIsCorrect()
      ```


    - #### setInitialisationNotCorrect

      ```
      public void setInitialisationNotCorrect()
      ```


- Overview
- Package
- Class
- Use
- Tree
- Deprecated
- Index
- Help

- Prev Class
- Next Class

- Frames
- No Frames

- All Classes

- Summary:
- Nested |
- Field |
- Constr |
- Method

- Detail:
- Field |
- Constr |
- Method
